# Sensory Neuromodulation

**DOI:** 10.3389/fnsys.2020.00012

**Published:** 2020-03-06

**Authors:** Robert D. Black, Lesco L. Rogers

**Affiliations:** Scion NeuroStim, Raleigh, NC, United States

**Keywords:** neuromodulation, noninvasive, sensory networks, brain oscillators, neurological disorders, neurovascular coupling

## Abstract

We describe a model of neurological disease based on dysfunctional brain oscillators. This is not a new model, but it is not one that is widely appreciated by clinicians. The value of this model lies in the predictions it makes and the utility it provides in translational applications, in particular for neuromodulation devices. Specifically, we provide a perspective on devices that provide input to sensory receptors and thus stimulate endogenous sensory networks. Current forms of clinically applied neuromodulation, including devices such as (implanted) deep brain stimulators (DBS) and various, noninvasive methods such as transcranial magnetic stimulation (TMS) and transcranial current methods (tACS, tDCS), have been studied extensively. The potential strength of neuromodulation of a sensory organ is access to the same pathways that natural environmental stimuli use and, importantly, the modulatory signal will be transformed as it travels through the brain, allowing the modulation input to be consistent with regional neuronal dynamics. We present specific examples of devices that rely on sensory neuromodulation and evaluate the translational potential of these approaches. We argue that sensory neuromodulation is well suited to, ideally, repair dysfunctional brain oscillators, thus providing a broad therapeutic approach for neurological diseases.

## Introduction

Our central aim is to introduce the concept of using sensory neuromodulation to improve the function of brain oscillators affected by the disease. (1) We start with a background on the premise that brain function is mediated by oscillatory dynamics and that, therefore, the neurological disease can be viewed as being associated with dysfunctional oscillators. (2) Next, a short review of the types of neuromodulation used clinically and a description of sensory neuromodulation, more particularly, is provided. (3) We then consider how brain oscillators maintain their stable function and how that changes with disease onset. (4) We then provide a detailed evaluation of vestibular neuromodulation (VNM), as an example of sensory neuromodulation, and clinical results obtained using time-varying caloric vestibular stimulation (CVS). (5) We conclude with a consideration of how specific neurological diseases might be approached using sensory neuromodulation methods, including the need for the measurement of parameters to guide titration. By necessity, we must consider a wide range of results from different fields in order to justify the relevance of an oscillator-centric view of neurological disease. The advent of non-invasive neuromodulation devices that work to modulate brain oscillators provides a clinically meaningful context.

### Goals of Neuromodulation

The term “neuromodulation” has multiple meanings for different audiences. Here, we focus on non-invasive devices that stimulate one or more regions of the brain using various stimulation methods to alter neuronal firing dynamics in the brain (examples of such devices are discussed below). We discuss devices that are designed to deliver therapy, vs. devices that perform diagnostic assessments. As described in detail below, the role of neuronal oscillations in theories and models of brain function, studies of consciousness, etc. is well established and widely accepted. However, clinical medicine does not typically frame brain disease in the context of dysfunctional brain oscillators (epilepsy being one notable exception). Instead, the primary focus is usually given to biochemical pathways and processes. Yet the aim of non-invasive brain stimulation (NIBS) devices is to change the state of brain oscillators, ideally returning them closer to a pre-disease state, and specific effects on biochemistry are not always a central consideration. We provide one detailed example where changes in brain oscillations mediate the activity of a well-known neurotrophic biomolecule (IGF-1), which helps to provide evidence that an oscillator-centric view of the neurological disease can be productive. That is, one can view the activity of brain oscillators as “commanding” some underlying biochemical processes (and not vice versa). As a preliminary, we now provide some background on oscillators in a biological context and in the human brain in particular.

**GRAPHICAL ABSTRACT:**
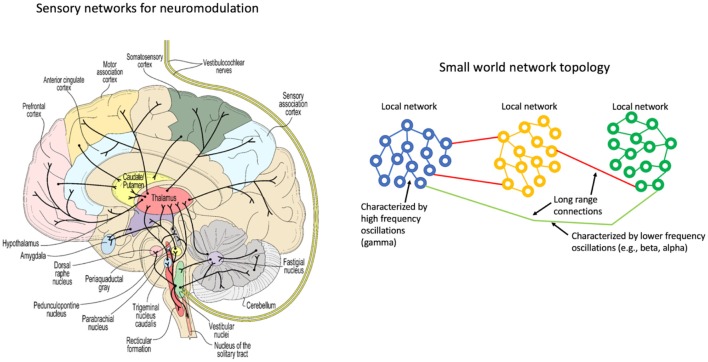
A network representation of the vestibular system (left) and a schematic representation of coupled oscillators in a small world topology (right).

## Oscillatory Dynamics

There is now growing evidence that brain dynamics are underpinned by collective oscillatory states. We explore the proposal that neurological disease can be modeled as dysfunctional brain oscillators. We further consider how artificial neurostimulation methods, using non-invasive devices, might alter brain oscillators and the potential for improving functional deficits resulting from disease. Most particularly, we examine neurostimulation introduced into an endogenous sensory network and examine how this approach is different from current clinical methods of neuromodulation.

### Neuronal Oscillations

The observation of neuronal oscillations has been documented in relatively simple animals such as aplysia (Elmariah, 2007) and jellyfish (Nath et al., 2017). Electroencephalography (EEG) recordings have been reported in eels (Barthélémy et al., 1977), fish (Robb and Roth, 2003) and reptiles (De Vera et al., 1994). De Vera et al. (1994) suggested a homology between the waking state of reptiles and slow-wave sleep in mammals. This is an evocative suggestion, but the primary finding is that oscillations enable behavior in animals in a manner that cannot be deduced based simply on the static architecture of the connectome.

Though EEG recordings have been studied for decades since the original work by Berger in the 1920s, their significance was not immediately understood. Until recently many researchers were unsure of whether the oscillations recorded in the EEG time series were meaningful or simply epi-phenomena. Fries (2005) proposed that oscillatory activity in the brain is actually central to function, enabling a means by which transient pathways can form and fade, based on demand. His communication through the coherence model provides one answer to the question of how the dynamic organization of the cortex occurs. More recently, McLelland and VanRullen (2016) reviewed communication-through-coherence and refinements.

Buzsáki’s (2006) comprehensive book takes a consistent, oscillation-centric perspective on the primacy of oscillations to brain function. He starts with a general review of periodic, nonlinear and chaotic phenomena in nature and from that develops a framework for understanding coupled oscillators and detailed results from invasive neuroscience studies before turning to non-invasive methods used in human research, including EEG, MEG, and functional imaging. He paints a story of continuity, both evolutionarily and architecturally, from small clusters of neurons to the whole brain. Voytek and Knight (2015) suggested that dynamic network communication relies on coordination *via* neuronal oscillations, the disruption of which can result in clinical disorders. Assenza et al. (2017) and Fox et al. (2014) reiterated the view that dysfunctional brain oscillators are associated with disease and they review how neuromodulation may be helpful in altering and improving brain oscillator function.

**Figure 1:**
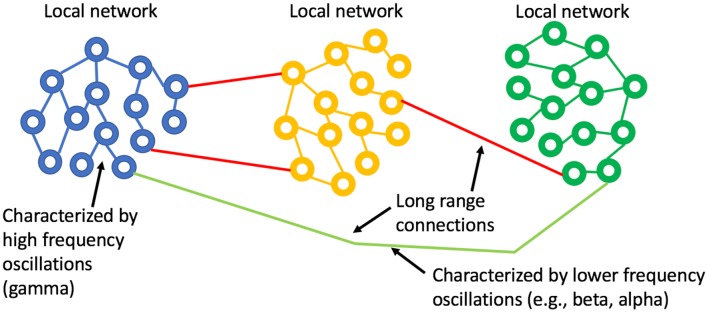
Small world network topology. The highest density of synaptic connections is local and longer-range connections are sparse. This topology is consistent with high frequency, short-range oscillations (gamma) being local and longer range, slower oscillations being regional or global.

There is significant literature that has applied oscillator models to biological systems or rather has attempted to understand biological processes with simplified oscillator configurations. Winfree (2001) and Kuramoto (1984) looked at the phenomenon of synchronization of biological oscillators, providing mathematical models for quantifying when synchrony can occur and what that may imply for studies of actual neuronal networks. Watts and Strogatz (1998) showed that the collective dynamics of so-called “small-world” networks could be used to explain the collective pathways observed in the brain. The essence of small-world topology is that most connections are local and sparse long-range connections allow for global communication (Figure 1). This form seems very well matched to the observation that gamma oscillations enable localized neuronal dynamics and slower (alpha, beta, etc.). EEG frequency bands are indeed associated with longer-range dynamics. The small-world topology is an optimal configuration for the efficient use of space (synaptic density) while still enabling global communication.

### Cross Frequency Coupling (CFC)

**Figure 2:**
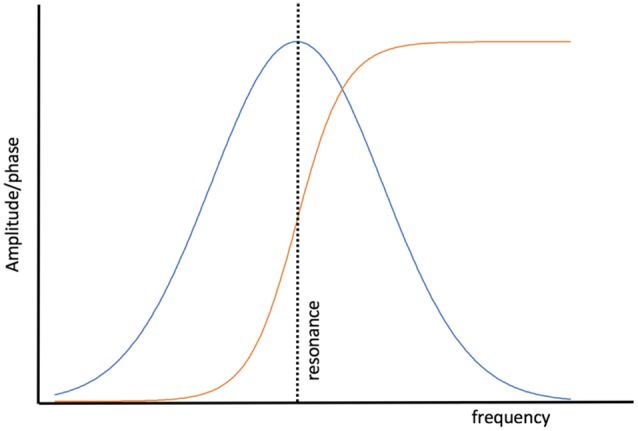
The behavior of a simple, undamped oscillator as a function of the excitation frequency. The amplitude of the response of the oscillator (blue) to the driving force peaks when the driver reaches the natural resonance of the oscillator. The phase of the oscillator with respect to the driver (orange) is in a phase when the driving frequency is lower than the resonant frequency and anti-phase when the driving frequency exceeds resonance.

**Figure 3:**
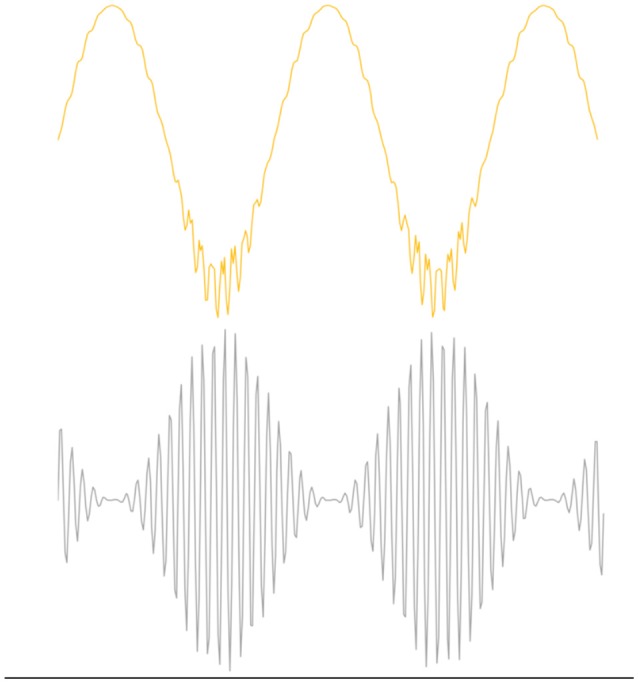
Example of Phase Amplitude coupling, one form of cross frequency coupling (CFC). The top figure illustrates the effect of coupling on beta oscillations where the bottom figure represents gamma oscillations. Beta modulates the amplitude of gamma and gamma perturbs the envelope of beta. CFC is essential for normal brain function, but over or under coupling can be pathological.

Entrainment of an oscillator at its natural frequency means that there is energy transfer between the driving or exciting oscillation and the target oscillator (Figure 2). An everyday example is a way a child “pumps” a swing until the natural frequency of the swing is reached and thereafter little additional effort is needed to maintain entrainment. A small but consistent excitation source is able to entrain an oscillator near its natural frequency and this efficiency is realized in brain oscillators as well (e.g., Buzsáki, 2006). *Via* a phenomenon called cross-frequency coupling (CFC), it is also possible to entrain oscillators that do not have the same natural frequencies. Instead, CFC describes the coordination of different oscillators whereby both oscillators have modified dynamics. Figure 3 shows an example of CFC between a fast and slow oscillation. The slower oscillation modulates the amplitude of the faster, and the faster makes ripples in the slower. This behavior has been recorded in brain oscillators and examples of aberrant coupling have been found as a result of the disease. For example, excessive CFC between gamma and beta frequencies has been documented in Parkinson’s disease (PD) patients with motor dysfunction (de Hemptinne et al., 2015). There are several forms of CFC that may have analogs in brain dynamics (Aru et al., 2015). CFC underpins the long-range connections described by the small-world network model.

**Figure 4:**
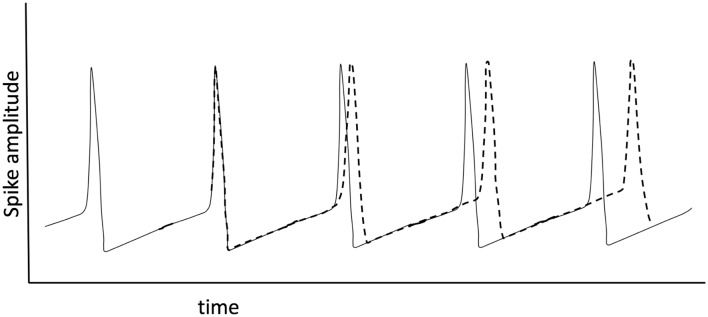
Spiking of a relaxation oscillator (the basis for the Hodgkin-Huxley model), where an applied, continuing perturbation shifts (dotted line) the phase of the spike train going left to right. This shift can be viewed as a change in spike timing, phase modulation, or frequency modulation: they are all equivalent mathematically.

The formation of dynamic small-world ensembles is underpinned by developmentally established neuronal pathways, but the transient selection of certain subsets of all possible pathways must rely on an encoding scheme. How might encoding and recall be enabled in an oscillator-based model? Hoppensteadt and Izhikevich (1998) provide a simple yet general schema that models thalamocortical interactions *via* weakly coupled oscillators and they develop an intuitive framework in analogy to FM radio principles. A primary insight from their model is that a cortical oscillator may participate in different ensembles by changing its frequency without changing the strengths of synaptic connections. The authors also clarify the point that the terms “inter-spike intervals,” “frequency modulation,” and “phase modulation” all describe the same thing, in the neuroscience, electrical engineering, and mathematical physics literature respectively, even though that unity of description is not generally appreciated (Figure 4). The crucial observation is that frequency and phase encoding are both biologically plausible as a means for creating the sort of transient neuronal ensembles that are consistent with oscillator-based theories of brain function. The particulars of how frequency and phase encoding are instantiated in real brain networks are not yet fully understood, but established analytical methods from past studies of oscillatory networks provide a fertile basis for model generation.

To summarize, can we view neurological disease as *resulting from* dysfunctional brain oscillators? The test of any model is whether it has predictive power and, clinically, whether it informs the delivery of therapy; and so we can now ask whether it might be possible to interface with and alter oscillatory brain networks in order to achieve a clinical benefit. Understandably, this is a very different therapeutic perspective from one based on looking for discrete biochemical pathways and trying to alter them, selectively, using an exogenous pharmaceutical agent. How would one know which oscillators to affect and how would such an interaction provide a benefit, vs. being neutral or negative in effect? We will survey the concept of sensory neuromodulation as a distinct format that may be particularly well matched to the challenge of interfacing with brain oscillators. Additionally, we will look at some evidence that suggests that taking an oscillator-centric view of a neurological disease might also provide insights into innate protective pathways in the brain that orchestrate underlying biochemical processes. That is, it may be that the brain is able to marshal a biochemical response to repair and maintain neurons in response to the aberrant firing patterns of damaged brain oscillators.

## Methods of Neuromodulation

As stated previously, the term neuromodulation can have a broad meaning, including pharmacological interventions designed to alter neuronal dynamics. Here, we concentrate on NIBS, which includes a class of devices that use externally placed electrodes to direct electrical current into the brain (usually cortical) tissue, that current being direct or alternating, or created by a rapidly changing magnetic field that induces current flow in the brain. The latter method, transcranial magnetic stimulation (TMS), does not require direct contact with the head. We will not try to summarize the vast NIBS research literature, however, we note that there are several recent reviews of clinical neuromodulation (see the Workshop summary under Bain et al., 2015, and references therein). NIBS has been used extensively in a research context and there has been translation to clinical medicine as well. TMS has been cleared by the FDA for major depressive disorder (Connolly et al., 2012) and for migraine headaches (Conforto et al., 2014). Two other alternating current methods have been cleared for the treatment of migraine headache, one using an applicator on the neck for stimulation of the vagus nerve (Silberstein et al., 2016) and another applying current to the forehead (over a branch of the trigeminal nerve; Schoenen et al., 2013). Deep brain stimulation (DBS) describes a category of interventions seeking to alter regional neuronal activity *via* a surgically implanted electrode powered by an implanted pulse generator. DBS, especially for movement disorders associated with PD, is a well-established clinical approach, but it is typically reserved for late-stage disease because of the invasive nature of the placement procedure and the concomitant high cost (Umemura et al., 2016).

Ali et al. (2013) summarized one particular challenge of matching an exogenously generated electrical stimulus to a target brain oscillator: “An important implication of this finding is that the frequency of applied stimulation should be matched to the frequency of the endogenous oscillatory state.…(and) the choice of stimulation frequency could represent a serious challenge as there is no clear preferred resonance or peak frequency.” We propose that neuromodulation of a sensory network addresses this matching challenge. If the brain target is accessed by endogenous neural (sensory) pathways and the modulation signal is applied to the sensory organ, the signal is transformed in a way that it is matched to the native dynamics of the target region. Sensory neuromodulation also opens the door to alternative means for delivering stimuli, such as using light (eyes) or sound (ears).

That a sensory system processes and transforms incoming stimuli is perhaps most intuitively understood by considering vision. The light pattern on the retina changes rapidly as the result of saccadic eye movements and these signals follow the optic nerve to the first visual cortical region (V1). The visual scene is broken into constituent elements, as was described in the seminal work by Hubel and Wiesel (1959) and further processing proceeds in a hierarchical fashion. Near the top of the visual hierarchy, the brain is able to maintain a persistent representation of a viewed object even though the input activity patterns from the retina itself do not match the activity patterns in that part of the cortex representing the viewed object (Wurtz et al., 2011). The retinal signals were transformed and combined with input from other brain regions with the net effect that a new dynamic oscillatory state consistent with the represented object emerged. What sensory modulation approaches have been studied? Visual, auditory, somatosensory and vestibular neurostimulation methods are described in the literature and we now list a few examples of clinical studies, with translational medicine intent, to better illustrate *sensory neuromodulation*.

### Visual Stimulation

A well-described method for modulating visual perception makes use of an optokinetic drum, a rotating cylinder with light and dark stripes that is viewed by a subject. Most commonly an optokinetic experiment aims to induce perceptions of self-motion and nystagmus. Kikuchi et al. (2009) performed optokinetic stimulation while acquiring blood oxygenation-level-dependent (BOLD) magnetic resonance imaging (MRI) data and demonstrated activation of cortical areas related to visual motion processing and deactivation of the parieto-insular cortex, which is primary in vestibular processing. Chokron et al. (2007) described studies using optokinetic methods for mitigating unilateral spatial neglect. The general aim was to correct spatial bias by realigning the perception of spatial coordinates. Those authors review a number of such studies and conclude that the effects were transient when single sessions were used. Kerkhoff et al. (2006) performed longitudinal optokinetic stimulations (using dynamic patterns on a computer monitor) and reported persistent improvement over a 2-week follow up period, which may imply a neuroplastic change as the basis for durability. Iaccarino et al. (2016) presented evidence in support of the reduction of plaque formation in a murine model of Alzheimer’s disease *via* optogenetically driven modulation of interneurons in the gamma frequency range. This is an interesting case wherein changes in oscillatory stimulation trigger a protective biochemical response. Using neuromodulation to activate neuroprotection is a topic to which we will return later.

### Auditory Stimulation

Bellesi et al. (2014) evaluated the possibility of enhancing slow-wave sleep (during non-REM sleep) using acoustic stimulation. They targeted modulation of a peripheral evoked slow-wave (K-complex) using entrainment *via* auditory stimulation. They list parameters of relevance for acoustic stimulation: intensity, frequency, timing (with respect to the onset of slow-wave sleep) and entrainment. The latter parameter is not independent of the others and instead speaks to the goal of matching the acoustic stimulus frequency to that of an endogenous oscillator. Acoustic stimulation at 0.8 Hz (that is, audible tones delivered at that rate) fits with the EEG power band of 0.5–1.0 Hz associated with slow-wave sleep and has resulted in higher intensity, suggesting entrainment of what is thought to be a spontaneous thalamocortical oscillation. A remaining challenge for this approach is to time the administration of the acoustic stimulus since stimulation at the wrong time in the sleep cycle can actually have an arousal effect. The authors suggest the use of ambulatory EEG to assess the proper time for administration, but this requirement increases the complexity of the method.

### Somatosensory Stimulation

Somatosensory neuromodulation is a challenging format to assess since the receptive organ can be the entire surface of the body. Wildenberg et al. (2010) take a novel approach and use the tongue for the introduction of stimulation *via* an array of contact electrodes (PONS device—portable neuromodulation stimulator). One significant advantage of this approach is that the wet, salty surface of the tongue provides a low electrical input impedance, obviating the need for cleaning the skin and applying electrode gels, as is common with transcutaneous current techniques. The tongue also has a high density of somatosensory receptors, as can be seen in images of the somatosensory homunculus, originally created by Penfield (Schott, 1993). The authors assert that tongue stimulation results in less non-specific brain activation as compared with other NIBS approaches. In Wildenberg et al. (2011), the authors used BOLD MRI to infer that tongue stimulation results in pontine neuromodulation, *via* the trigeminal nerve, and it interfaces with the balance-processing network. This non-intuitive discovery underlies the clinical focus of the tongue stimulator for balance disorders.

Wildenberg et al. (2013) hypothesized that the afferent output from tongue stimulation enters the brainstem in proximity to the vestibular and trigeminal nuclei, moving upwards to the cortex, and is able to influence cortical processing of visual motion. Leonard et al. (2017) undertook an imaging study with multiple sclerosis subjects to assess the effects of PONS stimulation over a 14-weeks treatment period. They found evidence of improved motor performance in active-arm subjects, as judged by BOLD MRI data localized to the motor cortex, suggestive of a neuroplastic change (specifically, the changes were durable enough to be subsequently recorded with functional magnetic resonance imaging, fMRI). Of relevance here is that the mechanism of action of the PONS device seems to involve extensive pathways, accessed *via* a sensory input channel, albeit an indirect one *via* the tongue and that there is evidence of durable neuroplastic change with beneficial effects for subjects (without significant device-related adverse events). A crucial observation is that this approach to sensory neuromodulation enables stimulation of brainstem regions. But the modulatory effects progress up from the brainstem to areas including the visual cortex and parieto-insular vestibular cortex (Wildenberg et al., 2013); and, importantly, the modulation signal follows endogenous sensory pathways.

### Multi-modal Stimulation

Marks et al. (2018) evaluated the application of simultaneous auditory and somatosensory stimulation as a treatment for tinnitus. Starting with a guinea pig model, the authors found that fusiform cells exhibited increased spontaneous activity and cross-unit synchrony, which are physiological correlates of tinnitus in a majority of patients. Through empirical means, they found that bimodal (but not unimodal) stimulation produced a long-term depression in the dorsal cochlear nucleus in guinea pigs. The stimulus method consists of sound stimuli, delivered by inserted earphones, and somatosensory stimuli, delivered by electrodes placed on the skin of the cervical spine or cheek. The auditory stimulus was based on the individual subject’s tinnitus spectrum and audiogram. The electrical (somatosensory) signal was timed to have a specific temporal relationship with the auditory signals (again, based on empirical findings in the guinea pig model). For human subjects, the device was designed for home use, thus facilitating daily, 30-min treatment sessions for two, 4-weeks sessions (with a gap) before being crossed over to the opposite treatment arm (active or placebo). Ten of 20 subjects had clinically significant reductions in their tinnitus scores. This study is an example of the use of two forms of sensory neuromodulation in an interactive protocol. The availability of a well-developed animal model allowed for specific hypotheses about the mechanism of action to be developed. Since tinnitus is a sensory processing disorder, its alignment with a sensory neuromodulation approach is logical.

### Vestibular Stimulation

We now turn to vestibular stimulation and argue that this sensory channel presents an attractive conduit for sensory neuromodulation. CVS is a widely used diagnostic technique, in particular for the study of balance disorders, and was initially explained by Barany and Wittmaack (1911). The Fitzgerald-Hallpike (Fitzgerald and Hallpike, 1942) protocol for CVS is used routinely in the diagnosis of vestibular disorders. Galvanic vestibular stimulation (GVS) is a transcranial current method with the specific intent of creating a voltage bias across the two sets of vestibular organs by placing electrodes on the mastoid bone behind the ears (Fitzpatrick and Day, 2004). CVS and GVS, along with rotational methods, will be collectively referred to as VNM.

**Figure 5:**
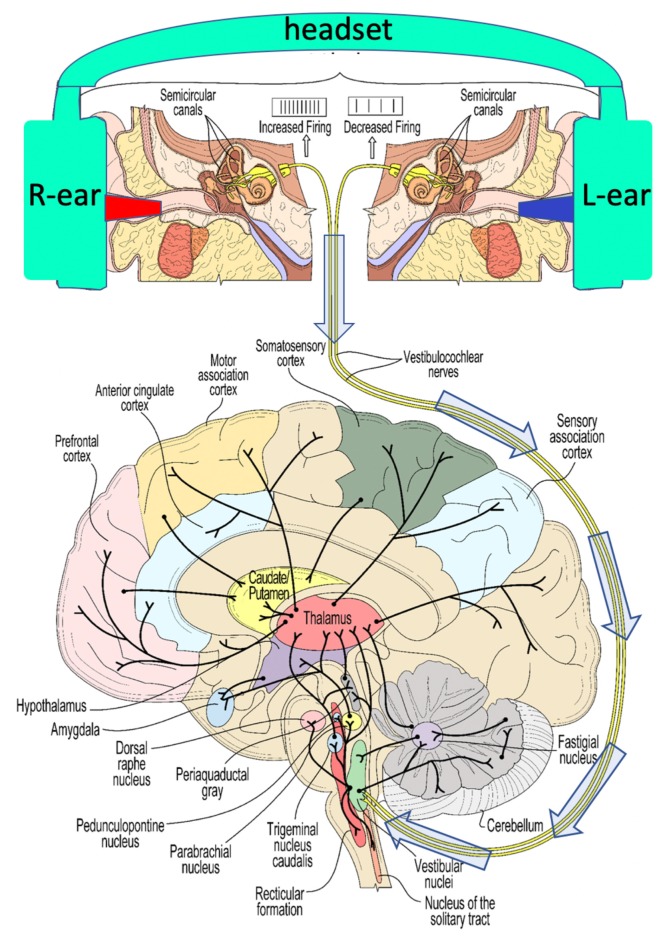
A schematic view of some of the widespread connectivity of the vestibular system. The top panel illustrates the induction of caloric vestibular stimulation (CVS) using warm (red)/cool (blue) ear inserts that are a part of a vestibular neuromodulation (VNM) device described below (see Figure 8 for example, thermal waveforms). The 8th cranial nerve conveys vestibular stimulation (arrows) to the vestibular nuclei in the brainstem.

The neuroscience of the vestibular system has been illuminated in extensive studies in animals and humans (Figure 5). Ayres’s (1972) book on the multi-sensory and integrative aspects of the vestibular system is a particularly cogent reference that illuminates the higher-order cognitive elements of vestibular processing, going well beyond the study of balance.

Klingner et al. (2012) reported on an independent component analysis of vestibular cortical function. Using BOLD (blood oxygen level-dependent) responses as markers of activity, seven different temporally defined components in the image data were identified. Four of these components were reported with a positive BOLD response. The authors stated that those components comprised the insula and retroinsular/parietal regions, the inferior/middle frontal gyrus, the superior temporal gyrus, the temporoparietal cortex, the parahippocampal gyrus, the hippocampus and the cerebellum. Three components were mainly characterized by negative deflections of the BOLD signal: the pre- and postcentral gyrus, the anterior cingulate gyrus, the precuneus, the occipital lobe, and the supplementary motor area.

Lopez and Blanke (2011) reviewed the extensive literature around the structures and pathways comprising the thalamocortical vestibular system. The authors concluded that there is no unique and well-defined primary vestibular cortex comparable to the primary sensory cortex for vision, somatosensation, and audition. The vestibular system is often referred to as a multi-sensory sense since it has direct or indirect projections into all cortical regions and vestibular sensory flow impacts the interpretation of other sensory modalities in the brain; and thus in addition to mediating balance, emerging evidence suggests that the vestibular network expands into dimensions of emotional processing, mental health and social cognition (Lopez, 2016).

Hitier et al. (2014) addressed the role of vestibular pathways in cognition, focusing on five major pathways that transmit vestibular sensory information to the distributed vestibular cortex: (1) vestibulo-thalamocortical; (2) dorsal tegmental nucleus *via* the lateral mammillary nucleus; (3) nucleus reticularis pontis oralis; (4) *via* the cerebellum, and (5) (hypothesized) *via* the basal ganglia. The cerebellum evolved from the vestibular and trigeminal nuclei (Bishop, 1959) and thus has a key role in processing vestibular sensory flow.

The cerebellum has, in the past, been relegated to a role in motor function, much as the vestibular system was viewed as being only relevant for balance. That view has now shifted to a more complete appreciation of this structure that contains four-times more neurons than the neocortex (Barton and Venditti, 2014). The cerebellum coordinates limb movement, controls movement-related sensory data acquisition, the timing of sensory acquisition and the prediction of the sensory consequences of action (Manto et al., 2012). The cerebellum works with the vestibular system to interpret sensory inputs, creating forward models so as to enable reactions to changes in the environment (Sultan et al., 2012).

In summary, the vestibular system, and its distributed representation in the neocortex, presents a very appealing set of pathways for neuromodulation devices. There is not a major brain region, cortical or sub-cortical, that is not innervated by neuronal networks impacted by vestibular activity. Also, as noted previously, the innate processing of sensory signals enables distributed modulation within the context of local brain dynamics. As an example, Nishiike et al. (1996) found that irrigation based-CVS led to a reduction of the firing rate of the locus coeruleus (LC), whether the CVS increased or decreased the tonic firing rate of the vestibular hair cells. In other words, the modulation of the firing rate of the vestibular system was transformed and resulted in a wholly different firing pattern in the LC.

## Descriptions of Disease in an Oscillator Framework

As we have stated, clinical medicine does not typically frame neurological disease in terms of dysfunction in brain oscillators. Therefore, let us look at some specific examples where a neurological disease may be viewed as resulting from the dysfunction of oscillatory states and evaluate whether that perspective generates any new insights.

Epilepsy is often viewed simply as being characterized by hypersynchronous seizures, but the ictal and inter-ictal patterns of synchronization are more complicated than that. Müller et al. (2014) described varying degrees of synchronization prior to and during a seizure event. They described the peri-ictal evolution of brain network function as transitioning from a predominantly random topology to a more regular network and back again. They suggested that high synchronization at the end of a seizure is, in fact, a signature of the termination of the seizure. Yet throughout, they stated that there is a consistent trace of topology associated with the default mode network (DMN). In other words, the default organization is not lost during a seizure and ultimately reasserts itself. Perhaps counter-intuitively, epilepsy seems to be characterized by cortical regions that have poor connectivity and the hypersynchronous activity of a seizure works to re-integrate disconnected regions (Rummel et al., 2013; Schindler, private communication). Kuśmierczak et al. (2015) addressed changes in local and long-range connectivity between cortical neurons as a component of the epileptogenic process after deafferentation in an animal model. They found evidence that as axons started to reconnect the transected regions, the balance of long- and short-range excitatory connections altered neuronal excitability. It may be that a similar process occurs during post-traumatic epilepsy or that similar imbalances occur with idiopathic epilepsy. Is a seizure the brain’s attempt to re-establish normal cortical connectivity?

Finding a biomarker for migraine onset is a current aim in headache research. Coppola et al. (2012) provided evidence that, interictally, migraineurs exhibit poor sensory habituation. Goadsby et al. (2017) argued that migraine headache is a disorder of sensory processing, which cycles based on development (genetics) and the environment. They focused on abnormal brainstem function in the premonitory phase of a migraine when the well-known sensory phobias emerge. Brighina et al. (2009) provided evidence that cerebellar inhibition is reduced in migraineurs and it is known that the cerebellum exerts inhibitory control on the cortex. Since the brainstem, cerebellum and cortex are all involved in sensory processing, a failure of habituation would seem to be a result of widespread network dysfunction. Interestingly, as the migraine develops, sensory habituation normalizes (Coppola et al., 2013). Is a migraine the brain’s attempt to re-establish normal sensory sensitivity?

Motor dysfunction in PD results from neurodegeneration, most particularly in dopaminergic pathways involving the substantia nigra and striatum. de Hemptinne et al. (2015) described an elegant experiment undertaken with PD patients who had implanted DBS devices and who were undergoing invasive brain surgery. This allowed the authors to use electrocorticography to measure cross-correlations between activity in the gamma and beta bands. PD patients receive DBS implants to mitigate some movement disorder symptoms and it had been suspected that over-coupling between beta and gamma in the motor cortex increased with the severity of the disease. Indeed, de Hemptinne et al. (2015) found that CFC increased with the DBS device turned off, and decreased when the device was active. How the neurodegenerative effects of PD altered the normal amount of CFC is not wholly clear, but this is a case whereby changes in normal brain oscillator function have a clear and measurable clinical consequence. As a result of neurodegeneration, the brain is not able to re-establish normal connectivity, but through artificial stimulation, it is possible to reduce pathological CFC.

In these three examples, epilepsy, migraine and PD, we see that the diseases are characterized by widespread network dysfunction, which implies collective action of brain oscillators if one accepts that healthy brain function is underpinned by collective oscillations. Taking an oscillator-centric viewpoint leads to a generalized way of understanding neurological diseases that are typically not considered together (though migraine and epilepsy are sometimes discussed jointly in terms of cortical spreading depression). Further, there is evidence for migraine and epilepsy that the brain acts to re-establish a more stable network configuration, but that allostatic drive seems to be associated with a seizure (in the case of epilepsy) or a migraine headache and therefore the brain’s response seems associated with the signal pathologies of the diseases. The use of DBS for motor dysfunction in PD suggests that direct alteration of aberrant oscillatory states can be therapeutic and we shall argue that sensory neuromodulation is particularly well-suited as a therapeutic methodology in this regard.

### What Keeps Oscillators Functional?

Adult neurogenesis may occur to a limited degree in the hippocampal complex (Anacker and Hen, 2017), but changes in existing neuronal structures in the adult brain are largely the result of neuroplastic alterations in networks; in synaptic connectivity. Adult learned behavior and memory formation can only occur through synaptic modification. Alterations in connectivity as a result of stroke have historically provided a significant source of understanding about how function is enabled by specific brain regions (Hallett, 2005). One particular observation with stroke patients provides an interesting conceptual model for functional loss more generally. An idling neuron (Neubauer et al., 1992) is a term that was coined to describe a neuron found in an ischemic penumbra with a living soma, but with a reduced dendritic arbor or overall reduced metabolism. The idling neuron is not dead, but it is not part of a network and therefore not functional. Within this model, early stroke intervention improves the likelihood of the re-integration of the idling neuron into a functionally useful configuration. One can see an analogy with neurodegenerative disease, where a neuron may be disconnected, but still alive. More generally, what innate processes maintain neural networks that underpin oscillatory brain states?

What keeps oscillators from failing or drifting to a new configuration? The extensive studies of the DMN bear on this question (Fox and Raichle, 2007; Raichle, 2015), but the answer is still unclear. Clearly, the “default” mode must be a stable configuration upon which intentional, task-positive functioning relies and thus should be resilient to perturbation or damage (Dosenbach et al., 2007). Pinal et al. (2015) considered age-related changes in the DMN by studying brain oscillatory activity in healthy young and old adults during a visual task. They found that whereas an age-related loss of synchronization occurred in executing the task, the default mode maintained synchronous activity, which they aptly describe as being stuck in the default mode. Aerobic fitness is consistently associated with the maintenance of optimal brain function. Talukdar et al. (2018) examined the brain connectome of healthy young adults, citing enhanced neuroplasticity in specific targeted regions. They found the benefits of aerobic exercise were indeed widespread, suggesting a primary causal relationship, but the study was not designed to speak directly to the mechanism of action. The role of sleep in maintaining stable brain networks is a subject of intensive study. Larson-Prior et al. (2009) concluded that the spontaneous BOLD MRI activity is seen in the descent to sleep reflect processes that maintain the functional integrity of the brain. They went on to note that even under general anesthesia, default functional connectivity remains intact. (If this were not the case, how would the patient re-emerge from anesthesia with an intact mind?). The synaptic homeostasis hypothesis (SHY; Tononi and Cirelli, 2006) posits that synaptic strength weakens during sleep in order to, conceptually, preserve important neuronal connections and winnow out unimportant ones. This must obviously occur while maintaining the DMN and in fact, evidence supports the importance of sleep for proper DMN function (Gujar et al., 2010).

Turrigiano (2011) summarizes the concept of synaptic homeostasis and addresses the question as to why brain oscillators act to maintain a stable, developmentally achieved state. She notes that cortical networks can appear highly unstable with extensive positive feedback and remarks that it’s rather extraordinary that epileptic seizures are not the norm. Reviewing a large body of empirical evidence, Turrigianno concludes that neuronal circuits maintain firing around a homeostatic stable point; and when perturbed, the circuits seek to return to a baseline set of dynamic parameters. This homeostasis obviously involves balancing excitatory and inhibitory firing, which is achieved through control of synaptic strengths. How this balance is maintained is obviously a significant and complex question, but it is easy to see how disease processes can distort this balance. Recently, Ma et al. (2019) framed synaptic homeostasis in the language of criticality. Criticality refers to non-equilibrium dynamics, specifically when a system is undergoing a discrete change in configuration. The idea has been used in brain models addressing normal brain function. We cite this result because criticality can then, also, be used to characterize abnormal brain function and parameters measuring the critical state might guide the use of neuromodulation therapy and gauge its success.

Chan et al. (2016) considered correlates between neuronal oscillations and mitochondrial dysfunction. In this case, the authors focused on the disruption of cortical oscillators due to the weakness of the neurons comprising those oscillators, resulting from metabolic stress *via* mitochondrial damage. The authors looked at a number of examples of diseases for which this linkage could have diagnostic and therapeutic import. We now return to the idea that changes in baseline oscillator function may trigger innate neuroprotective responses. Might the brain use changes in oscillators to trigger biochemical responses that work to “fix” dysfunctional oscillators? We consider one such example below.

### An Example of Innate Neuroprotection

**Figure 6:**
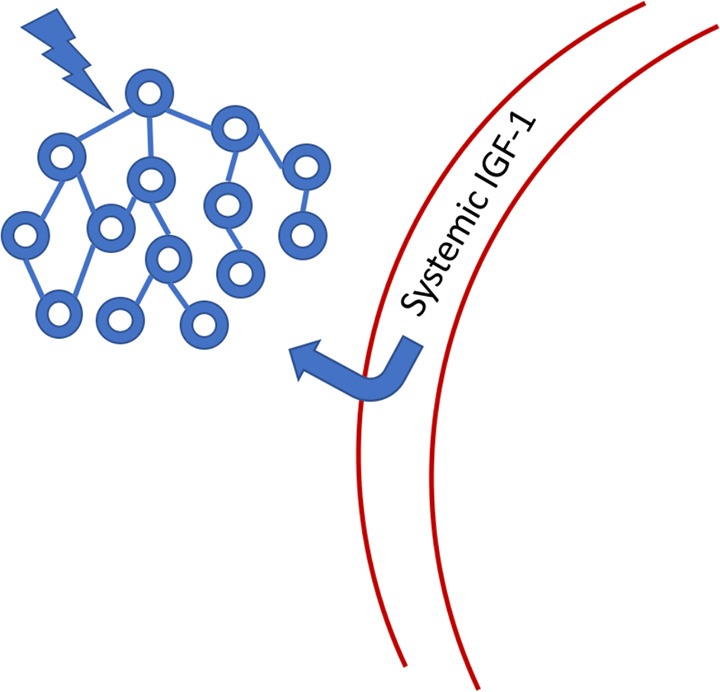
Systemic IGF-1 is transported through the blood-brain-barrier (BBB) to the site of neuronal activity. IGF-1 binding protein is cleaved from the IGF-1 molecule, allowing passage through the barrier. Once in the CNS, IGF-1 has a limited half-life because it no longer has its binding protein chaperone. See Nishijima et al. (2016) for a more detailed figure.

*The Torres-Aleman group* has proposed a compelling hypothesis that links oscillatory brain activity to the maintenance of the neurons that comprise such oscillators. We list some of the findings and predictions associated with this hypothesis as a preliminary to exploring how sensory neuromodulation might co-opt a natural process for maintaining the integrity of neural network function. In Nishijima et al. (2010), the authors detailed a pathway by which IGF-1 (insulin-like growth factor) enters the central nervous system (CNS) from systemic circulation in response to the activity of neural oscillators (Figure 6). IGF-1 is a very well-studied neurotrophic biomolecule that has been impressively stable across evolutionary time. It is reported to improve resistance to mitochondrial apoptosis (Kang et al., 2003; Gu et al., 2004; Akundi et al., 2012) and promote synaptogenesis (Nieto-Estévez et al., 2016). However, just increasing IGF-1 levels can lead to negative consequences for cell viability (Bitto et al., 2010). Therefore, for IGF-1 to be effective as a neuroprotective agent, it must be supplied when needed in response to neuronal activity or stress. Nishijima et al. (2010) showed that the activated process by which IGF-1 crosses the blood-brain-barrier (BBB) is frequency-dependent, suggesting that this pathway evolved to support and maintain baseline neuronal dynamics. They hypothesize that the pro-neurogenic effects of epilepsy, some forms of external neural stimulation, and exercise may all depend to some degree on activated transport of IGF-1 through the BBB. Nishijima et al. (2016) expanded the model of IGF-1 transport and emphasize that uptake is dependent on cerebrovascular availability of IGF-1 to cross the BBB. Since exercise is understood to improve cerebrovascular blood flow (CBF), there is a direct link to the enhancement of IGF-1 uptake.

We, therefore, see that the maintenance of stable oscillatory states in the brain is essential to proper function over time and this reality suggests that there are innate response mechanisms that support this stability. That neuronal dynamics regulate the transport of IGF-1 through the BBB provides a specific example of how a neurotrophic biochemical response acts to support neuronal health and function. Toth et al. (2015) reported on the disruption of neurovascular coupling (NVC) in a murine model resulting from IGF-1 deficiency, emphasizing the integrative role of neuronal activity and the metabolic function mediated by the vasculature (see Appendix for additional IGF-1 references).

In summary, our aim is not to suggest that *only* oscillators matter and that biochemical pathways are only controlled by changes in oscillators. Rather, there is clear evidence that oscillators “resist” change from their developmental states, they exhibit homeostatic properties, and in order to achieve homeostasis, they must depend on supportive biochemical responses.

## Vestibular Neuromodulation Therapy

**Figure 7:**
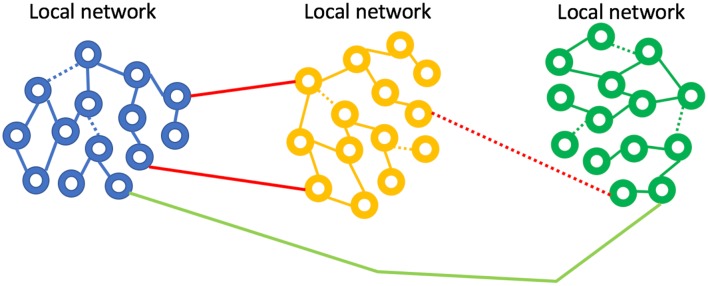
Neurological disease represented as weak or broken network connections. Sensory neuromodulation is hypothesized to force activity in weak networks, strengthening synapses and improving coupling. The IGF-1 mechanism may be a key player in this network repair process.

We now concentrate on one form of non-invasive sensory neuromodulation: vestibular. When applying artificial neuromodulation to a neuronal network, with the aim of altering synaptic connections, why would the effect be beneficial, vs. neutral or negative? Sensory neuromodulation delivers a signal, even if it is non-physiological, *via* an innate network that processes the artificial signal just as it would naturally occurring sensory stimuli. We propose, therefore, that sensory neuromodulation may act to encourage developmentally established network behavior and thus may act to strengthen endogenous oscillators that couple to the sensory network. In other words, the sensory traffic drives target oscillators in a manner consistent with innate function, consistent with the concept of synaptic homeostasis (Turrigiano, 2011). We hypothesize that sensory neuromodulation might then act to rehabilitate dysfunctional neural networks, bringing them back closer to a developmental state *via* neuroplastic modification (Figure 7). Evidence for such rehabilitative potential for sensory neuromodulation is preliminary, but we now review some clinical results that are consistent with this hypothesis.

CVS has been used diagnostically for decades, and there have been a number of small studies using CVS in a therapeutic context (e.g., Kolev, 1990; Rode et al., 2002; McGeoch et al., 2008, 2009; Grabherr et al., 2015), but diagnostic-style CVS devices, which use water or air to flood the ear canal, are not amenable to home use. Current GVS devices are also not easily adapted for home use because of the need to clean the skin carefully, apply a conductive gel, and affix electrodes over the mastoid bones behind the ears, but an even larger body of work exploring potential clinical applications of GVS exists (e.g., Yamamoto et al., 2005; Pan et al., 2008; Pal et al., 2009; Kerkhoff et al., 2011; Kim et al., 2013; Samoudi et al., 2015; Wilkinson et al., 2014; Okada et al., 2015; Hwang et al., 2016; Kataoka et al., 2016; Cai et al., 2018). The ability to conduct longitudinal VNM therapy is necessary to achieve sustained clinical efficacy and having a home-use device format is the most tractable approach to enable chronic therapy.

### Time-Varying CVS (tvCVS)

**Figure 8:**
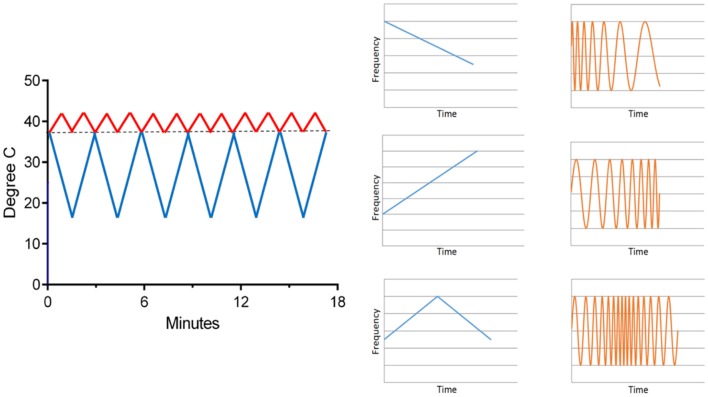
An example of time-varying waveforms used with the solid-state CVS device (left). One ear receives a warm triangle wave and the other a cold triangle wave. As seen in Figure 5, warming increases the afferent firing rate and cooling decreases it. On the right, the effect of a time-varying temperature on the hair cell firing rate is shown graphically. The response (orange) frequency shifts in time, a phenomenon called a “chirp.” Thus, a complex range of firing rate modulations can be achieved.

In order to extend treatment times with CVS, a time-varying thermal stimulus is needed to avoid adaptation of the vestibular hair cells (Bock et al., 1979) and so a solid-state CVS device (Figure 5; Black et al., 2016) was designed and equipped with the means to deliver time-varying thermal waveforms, independently to both ears (Figure 8). CVS alters the tonic firing rate, of ~100 Hz, of the regularly firing vestibular hair cells. Applying a triangular temperature waveform (Figure 8) results in a time-varying firing pattern in the vestibular hair cells around the 100 Hz tonic rate. Additionally, the envelope of the triangular waveform establishes a slower modulation and when both ears are stimulated simultaneously, at different frequencies, the resulting afferent firing pattern reaching the vestibular nuclei can be quite complex and spans frequencies from 0.01 Hz to 100+ Hz. Therefore, even though the time course of thermal transfer to the inner ear during CVS is slow, at least on the order of seconds, it is important to recognize that modulation of the equilibrium firing rate means that frequencies centered on 100 Hz are also present.

### Migraine

The vestibular neuroscience of migraine is explored in Balaban et al. (2019). Time-varying CVS (tvCVS) demonstrated efficacy in reducing headache burden in episodic migraineurs (Wilkinson et al., 2017). Migraineurs used the CVS device on a daily basis, at home. Over a 3-months long treatment period, a per-protocol reduction of 3.9 migraine days relative to a baseline of 7.7 migraine days. A control group showed a 1.1-day reduction from a baseline of 6.9 days. Analysis indicated that blinding was effective. Subjects used up to one concurrent preventive drug and as-needed abortive drugs and so the gains with CVS device use were additive, likely due to a parallel mechanism of action. This device was recently cleared by the FDA for use in treating episodic migraines in adults and adolescents.

The migraine study involved slow modulations in temperature, in the range of 0.008–0.033 Hz. The temperature changes then induce alterations in the firing rate of vestibular hair cells in the same range of frequencies. Is there any evidence that such slow oscillations might be important, on top of the basic need to avoid adaptation of the vestibular response? Black et al. (2016) provided evidence of entrainment of a pontine pacing center, which engendered oscillations in cerebral blood flow velocity, using tvCVS. In that study, the oscillations appeared to sharpen to what was presumably a natural resonance, at about 0.025 Hz and persisted when CVS was stopped, a strong indicator of entrainment. Sliwka et al. (2001) reported that migraineurs had abnormal B wave activity (interictal), spontaneous oscillations in blood flow velocity in a range from 0.008 Hz to 0.05 Hz. B waves are thought to originate in the pons and may be part of a the autoregulatory response (the B wave period is roughly the transit time of blood from the heart to the brain and back). Sliwka and colleagues suggested that abnormal B wave activity in migraineurs may stem from a dysfunction in the monoaminergic/serotonergic system in the brainstem, a hypothesis that overlaps independent models of migraine pathogenesis (Coppola et al., 2009). Therefore, evidence for entrainment of B waves with tvCVS could be a biomarker of utility when seeking to titrate therapy for individual migraineurs.

Oscillations in the B wave frequency range have also been observed in BOLD MRI studies aimed at measuring cortical functional connectivity (Cordes et al., 2001; Leopold et al., 2003; Schmidt, 2009; Bharath et al., 2017) and appear in slow-wave sleep (Dang-Vu et al., 2008). Dang-Vu et al. (2008) suggested a possible relationship between the observed oscillations in slow-wave sleep and waking DMN, implying a restorative role of sleep on large-scale cortical functional organization. Oscillations in the B waves range during sleep have also been seen with EEG (Terzano et al., 1985) and with transcranial Doppler sonography in newborns (Ferrarri et al., 1994). Are the oscillations seen in functional connectivity studies related to those seen in cerebrovascular studies or is there just a coincidental overlap in frequency ranges? Even if the two phenomena do not share a common pontine pacing center, entraining one, sets up the possibility of entraining the other. Measuring the onset and strength of entrainment of targeted brain oscillators presents a tangible method for the titration of therapy. No functional brain oscillator can remain isolated (uncoupled) from other oscillators (this is the essence of the small-world network concept) and tvCVS presents a powerful method for exciting *complete networks*, innervated by the vestibular system. Coupling occurs not just for oscillators that have the same resonant frequency, but cross-frequency coupling (CFC) allows for interactions between oscillators having different fundamental frequencies.

B waves might also be of interest in assessing the health of NVC. NVC has gained interest amongst researchers as a key factor influencing the progression of neurodegenerative disease. Iadecola (2017) stated that maintaining neurovascular health promotes brain health and Lecrux and Hamel (2011) looked at Alzheimer’s disease, in particular, in the context of alterations in neurovascular function. Toth et al. (2017) summarized the interplay between cerebral autoregulation, in which B waves play an important role and NVC. The authors suggested that understanding vascular contributions to cognitive impairment and dementia has significant implications for preserving brain function in older individuals. Ozturk and Tan (2018) examined the evolution of cerebrovascular function and its support for the unique functionality of the human brain. Their work highlights the inevitability of the effects of neurovascular pathology on cognition.

### Minimally Conscious State

tvCVS has also been used in a case study with two subjects in a minimally conscious state (Vanzan et al., 2017) with the aim of increasing awareness. Spontaneous recovery for these subjects generally occurs within a time window after emergence from a coma and both of the subjects were past the time when natural recovery was expected. As measured with coma recovery scales (WHIM and CRS-R), improvement seemed to show a time-locked association with CVS treatment epochs, which consisted of 1-month of daily treatment, followed by 1-month of sham treatment, and ending with the second month of active treatment. Improvements were recorded during active treatment and no gains were seen during sham treatment. Note that the subjects were likely unaware of the active/sham changes. One of the subjects, in particular, demonstrated durable gains and progressed from an inability to move his gaze to a person in the room to initiating conversation and answering simple questions. The authors noted that the apparent gains imply improved function across a number of brain regions, but most particularly the thalamocortical projection system, and neuroplastic modification of neuronal pathways. They further suggested that vestibular pathways go well beyond a role in autonomic motor control and underlie higher cognitive states.

### Parkinson’s Disease

**Figure 9:**
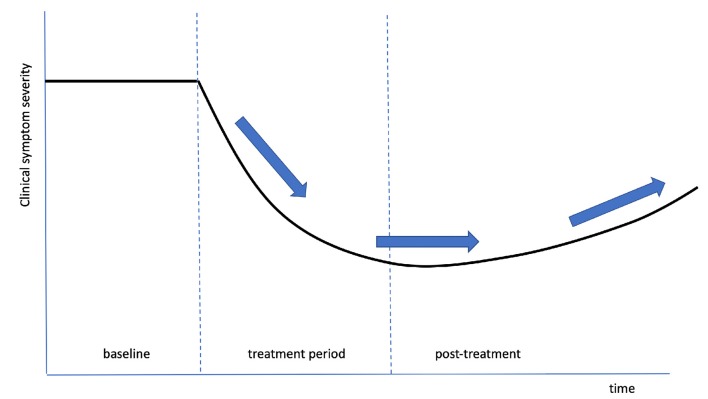
An idealized composite graph of the response, over time, of multiple, different symptoms in Wilkinson et al. (2019). The same characteristic shape and time course was seen across independent measures, suggesting a common effect, possibly neuroplastic modification of the associated dysfunctional pathways. The arrows show initial improvement, a period of durable gains, and a slow return towards the baseline.

As a final example of clinical results based on tvCVS therapy, we review work summarizing longitudinal therapy delivered to PD subjects. An initial case report (Wilkinson et al., 2016) found evidence of gains in several motor areas, with some evidence of durability past the end of treatment. That study was followed with a single-site RCT (Wilkinson et al., 2019) with the aim of measuring changes with both motor and non-motor scoring scales. All subjects were on dopamine replacement therapy (DRT) and treated daily for 2-months. They were evaluated in the “on” state (had recently taken their DRT medication). A blinding analysis concluded that subjects were unaware of whether they were in the active or placebo arm of the study. There are two general aspects of the study that stand out: broad efficacy across motor and non-motor symptoms and durability of gains for months after the end of treatment. When a DBS device is switched off, motor symptoms re-emerge almost immediately. There is no single drug that has shown efficacy for both motor and non-motor symptoms (Seppi et al., 2011). Figure 9 is derived from Wilkinson et al. (2019), amalgamating graphs from a number of different measures into one semi-quantitative graph. The same general time dependence appears, suggesting a slow return to baseline over a timeframe that seems too long to be explained by changes in up/down regulation typically seen in receptor binding kinetics (Zhuang et al., 2013). If the data are representative and if this result can be reproduced in a larger, multi-site RCT, then the most likely explanation is that multiple neuronal pathways were improved by the use of tvCVS, through neuroplastic modification.

## Discussion and Future Questions

We have reviewed the evidence supporting the contention that brain function is enabled by oscillatory activity of neuronal networks, at both local and global scales, and that these developmentally established oscillatory networks are actively maintained to ensure proper brain function. Therefore, one can propose a model of neurological disease based on specific changes in oscillatory networks. Using neuromodulation, *via* implanted or non-invasive means, to alter, and ideally correct, aberrant oscillation patterns is a compelling translational goal. We propose that sensory neuromodulation, defined as the use of endogenous sensory networks to deliver a neuromodulatory signal to the brain, is intrinsically extensive in reach and thus acts to modulate multiple oscillators systems in parallel and the modulation signal is transformed to be consistent with local neuronal oscillator dynamics. We suggest that modulating many brain oscillators in parallel is important for the clinical end goal of altering and improving network function, especially in the case of neurodegenerative disease. We have provided one specific example from the literature of how oscillator signaling can trigger biochemical responses *via* IGF-1, which acts to protect neurons and facilitate network function. Admittedly, sensory neuromodulation is in the early days of development, but there is already growing clinical evidence that it may have multi-faceted impacts on disease symptoms that is quite different from the generally narrow focus of pharmaceutical targeting (Drews, 2000), where the concern is to avoid non-specific targeting and adverse events. Much of our focus has been on tvCVS, based on the authors’ direct experience with the method, but also because the vestibular sensory network is so well-suited to the task of delivering neuromodulation throughout the brain. At present, the way in which sensory neuromodulation works, and how well it works, is not fully established and more clinical studies and mechanism of action studies are required. Yet it is reasonable to hypothesize, using the oscillator model of neurological disease, about what to expect.

### Why It Helps and Does Not Hurt

An oscillator-centric model clarifies how sensory neuromodulation can in principle work broadly and may mitigate multiple disease symptoms. This is because the nature of networked oscillators is to enable cross-coupling between individual oscillators and networks. It is for this reason that it behooves clinicians to consider oscillator-centric models when characterizing neurological disease.

As an illustrative example, consider the cardiac-oscillator. Heart rate variability (HRV) is a measure of the ability of the heart to respond to changes in demand from the body. High HRV means that the heart is very adaptive and is associated with good health. Low HRV means that the heart is not adaptive and means that the cardiac oscillator system is too weakly coupled to other brain-driven oscillator networks. If an oscillator is too weakly coupled, it is ineffective and thus the interventional goal is to improve and increase coupling to a normal level. In the case of the cardiac oscillator, exercise and a healthful lifestyle are well accepted as improving cardiac function, improving HRV (Kiviniemi et al., 2010). Aerobic exercise encourages the heart to be rate-adaptive and we submit that forcing the cardiac oscillator to be responsive to the demands of the body is achieved by exposing it to an appropriately stressful stimulus. Conversely, inactivity places low demands on HRV and the cardiac oscillator will become more weakly coupled to the body. The cardiac oscillator has a developmentally established level of coupling to the body, but the strength of coupling can change: it can improve *via* exercise or it can decline *via* inactivity. We assert that beneficial coupling is encouraged by forcing a target oscillator to work with other network oscillators and coupling is altered (Figure 7) through synaptic modification (neuroplasticity). This realization has important consequences for therapeutic intervention. First, it’s not possible to harm the function of a target oscillator by entraining it with other oscillatory networks, for, indeed, entrainment is fundamental to its function. Thus, oscillator entrainment by sensory neuromodulation will not interfere with normal function. The stimulus does not need to be focused only on a damaged region, for instance. Second, entrainment works to reinforce the integrity of an oscillatory network’s developmentally derived form and function (Turrigiano, 2011). We hypothesize that sensory neuromodulation provides input through endogenous channels and thereby forces functional responses, which reinforce synaptic coupling, in those areas innervated by the sensory pathways. It is for this reason that the extensive innervation of the vestibular network recommends it as a preferred conduit for sensory neuromodulation therapy. Understanding how sensory neuromodulation should work to maintain and improve coupling between oscillators would lead to the expectation of extensive effects on function (and thus potentially the mitigation of multiple disease symptoms) and durability of gains (because the effect is to create a neuroplastic modification of the coupling between oscillators).

The picture is not as simple as the one discussed above. If a neural network is disrupted by trauma or a neurodegenerative disease, then one can posit that the goal of sensory neuromodulation therapy is to re-established the pre-trauma, pre-disease configuration. The assumption is that the developmentally established network configuration is a preferred baseline to which the system seeks to return. But what about a disease that results in developmental abnormalities? In that case, there is no normative configuration to which to return; and diseases that have a strong behavioral component may not be amenable to therapy alone without some form of physical or cognitive behavioral therapy. Let us look at each of these three examples in turn.

A neurodegenerative disease (like PD) or disease that is trauma-induced (like TBI or stroke) results in an alteration in pre-disease oscillatory networks. Early evidence from Wilkinson et al. (2019) suggested that there may be a plastic response to tvCVS that works to return function broadly (possibly broad improvement of NVC). It may be that concomitant physical training could help with motor dysfunction, in particular, and this would result in a ratchet effect whereby neuromodulation improves function and physical therapy improves function further. Studies with the PONS device typically included a physical training component (Chisholm et al., 2014). An interesting question for future research is whether sensory neuromodulation creates neuroplastic facilitation that augments conventional physical therapy.

Diseases like schizophrenia and idiopathic migraine are developmental. If there is no disease-free baseline to which to return, what does this mean for the applicability of sensory neuromodulation? Gerretsen et al. (2017) used irrigation-based CVS with schizophrenia subjects and found evidence of transiently improved insight into illness. The authors concluded that similar to hemispatial neglect, CVS acted to balance activity in the two hemispheres, making use of the potential of unilateral CVS to preferentially activate one hemisphere. This is an interesting example as it points out the spectral nature of diseases like schizophrenia where improvement in one symptom may have a wider impact on patient outcomes. Wilkinson et al. (2017) offered clear evidence of benefit from tvCVS for idiopathic episodic migraine. Why this is so is unknown, but we noted earlier that the brain actually does try to re-establish normal sensory habituation during an attack; and so perhaps a helpful viewpoint would be to think of the migraine brain as having two states, habituating and non-habituating, and neuromodulation increases the prevalence of habituation, thus reducing the sensory dysfunction that may be the source of the disease (Goadsby et al., 2017).

For behaviorally driven diseases like type-II diabetes and addiction, it seems unlikely that neuromodulation alone cannot be effective since habits and lifestyle must also be adjusted. The aim, rather, for neuromodulation could be to make the adoption of lifestyle changes easier and to help to establish new habitual patterns.

Another challenge for sensory neuromodulation is how best to titrate therapy for a given individual. To meet this challenge, the establishment of suitable biomarkers would be helpful. As one example, EEG provides a measure of pernicious CFC in motor symptoms of PD (de Hemptinne et al., 2015). Black et al. (2016) provided evidence that tvCVS (and not constant temperature CVS) created cerebral blood flow velocity oscillations, possibly providing a target of relevance for migraines. Functional imaging studies do not lend themselves to routine applications for individual patients, but general lessons can be learned about how best to deliver sensory neuromodulation (Wildenberg et al., 2013). It may be possible to use inexpensive and non-invasive methods, like heart-rate variability, as a proxy for more elaborate procedures once the relevant parameters are identified. Looking for evidence of entrainment of a target seems like a fertile place to start. Ultimately, as we learn more about the mechanisms of action of sensory neuromodulation, we will understand better how to titrate therapy more effectively.

## Conclusion

The advent of home-use, low-risk devices for delivering sensory neuromodulation creates a distinct advantage in that such therapies can be used early in the progress of a disease, vs. implanted devices that are relegated to late-stage disease. This potential meshes with current efforts to detect neurodegenerative disease at an early stage, *via* the use of biomarkers. Taking therapeutic action simply on the basis of genetic profiling is a challenging decision, ethically, however a low-risk, low side-effect therapy mitigates the downside of acting early.

Viewing neurological disease in terms of dysfunctional oscillators enforces a systemic viewpoint since widely separate brain oscillators develop with the ability to interact with each other. Further, one can create a chain of reasoning by which the maintenance of default oscillatory dynamics is the guiding force that mediates some underlying biochemical responses that work to instantiate innate neuroprotection. We have suggested that neuromodulation *via* a sensory organ is a particularly attractive approach by which to improve the proper functioning of neuronal oscillator networks.

We have highlighted tvCVS, in particular, as a new form of sensory neuromodulation (and NIBS more generally), describing results from clinical studies suggesting that it may be acting broadly to improve dysfunctional brain oscillators and thereby improve functional connectivity. It is the case that an oscillator-centric viewpoint of the mechanism-of-action of tvCVS seems consistent with both broad clinical efficacy and durable gains observed clinically. Future studies will evaluate the specific hypothesis that tvCVS acts to improve NVC by promoting neuroplastic and cerebrovascular plastic improvement in brain regions compromised by neurological disease.

## Author Contributions

RB: primary and corresponding author. LR: secondary author. The authors jointly developed the hypotheses described in the manuscript.

## Conflict of Interest

Both authors are employees/shareholders in Scion NeuroStim, Raleigh, NC, USA.

## APPENDIX: Additional Igf-1 References

It is hard to overestimate the ubiquity of IGF-1 in metabolic processes. These references provide an illustrative, though very incomplete, overview: Sharma et al., 2016 and Ebert et al., 2008 [IGF-1, glial cell line-derived neurotrophic factor (GDNF), vascular endothelial growth factor (VEGF)]; Ng and Tang, 2013 [IGF-1 and sirtuin 1 (Sirt1)]; Barbieri et al., 2009 [IGF-1 and telomere length]; Ayadi et al., 2016 [IGF-1 and neuroprotection of striatum]; D’Mello et al., 1997 and Suzuki et al., 1998 [IGF-1 and inhibition of apoptosis]; Sonntag et al., 2013 [IGF-1 and cerebrovascular aging]; Zheng and Quirion, 2004 [IGF-1 and brain-derived neurotrophic factor (BDNF)]; Gontier et al., 2015 [IGF-1 and amyloid-beta]; Tran et al., 2014 [IGF-1 and cellular senescence]; Grinberg et al., 2013 [IGF-1, tumor necrosis factor-alpha (TNF-α), and oxidative stress]; Robida-Stubbs et al., 2012 [IGF-1 and target of rapamycin (TOR)]; Mason et al., 2001 [IGF-1 and interleukin-1β].) In terms of IGF-1’s role in disease progression, a small listing of studies of interest: Sung et al., 2014 [IGF-1 and amyloid beta in Alzheimer’s disease]; Grinberg et al., 2017 [IGF-1 inhibits cortical spreading depression]; Trueba-Sáiz et al., 2013 [loss of serum IGF-1 as early biomarker of AD]; Major and Valdivia, 2018 [disruption of IGF-1 homeostasis leads to neurodegenerative disease]; Yang et al., 2018 [IGF-1 signaling in Parkinson’s disease]; Aberg et al., 2006 [IGF-1 and neuroprotection in the adult brain]; Pitt et al., 2017 [IGF-1 and synaptopathy]; Levine et al., 2012 [IGF-1 and signal transduction in PD]; Sonntag et al., 2013 [IGF-1 and cerebrovascular aging].
